# Comparative diagnostic efficacy of serum squamous cell carcinoma antigen in hepatocellular carcinoma

**DOI:** 10.1186/1756-0500-5-403

**Published:** 2012-08-03

**Authors:** Olufemi Michael Soyemi, Jesse Abiodun Otegbayo, Samuel Olawale Ola, Adegboyega Akere, Temitope Soyemi

**Affiliations:** 1 Gastroenterology, Department of Medicine, University College Hospital, Ibadan, Oyo State, Nigeria; 2 Liver and Gastrointestinal unit, Department of Medicine, University College Hospital, Ibadan, Nigeria; 3 Liver and Gastrointestinal Unit, Department of Medicine, University College Hospital, Ibadan, Nigeria; 4 Liver and Gastrointestinal Unit, Department of Medicine, University College Hospital, Ibadan, Nigeria; 5 Department of Radiology, University College Hospital, Ibadan, Nigeria

## Abstract

**Background:**

Hepatocellular carcinoma (HCC) is a common liver malignancy in Nigeria. Hepatitis B and C viruses, alcohol and Aflatoxin B are among the various aetiologies. More work needs to be done in the search for markers that will aid early detection of this condition as it is uniformly fatal once advanced. Alphafetoprotein (AFP) remains the most widely used tumour marker of HCC detection in spite of its known shortcomings. The objective of this study was to determine the efficacy of serum squamous cell carcinoma antigen (SCCA) , in comparison to alphafetoprotein in the detection of HCC.

**Method:**

Sixty patients with HCC and thirty apparently healthy controls attending the Medical Outpatient Department(MOPD) of the University College Hospital Ibadan(UCH) Nigeria were selected for the study. Questionnaire was used to collect clinical data while AFP, SCCA levels, serum HBsAg and anti-HCV were determined using ELISA method- (Diagnostic Automation Inc. Canada). Abdominal ultrasound scan was also done.

**Result:**

Thirty one (51.7%) out of 60 selected cases were positive for HBsAg while six (20%) out of 30 controls were positive for HBsAg(p = 0.004). Out of the 60 cases selected for this study, only 2 (3.3.%) cases were positive for hepatitis C virus, while only 1(3.3%) out of 30 control was positive for hepatitis C virus(p = 0.74).

The mean AFP value for cases with HCC was 393.21 ng/ml ±386.97 compared to the control group which was 5.60 ± 13.03 ng/ml (p value 0.001). The mean SCCA level was 0.64 ± 0.56 ng/ml and 0.71 ± 0.65 ng/ml for cases and controls respectively (p = 0.631).

**Conclusion:**

Alphafetoprotein remains a good tumour marker for the diagnosis of HCC. Serum squamous cell carcinoma antigen(SCCA) has no discriminatory power and may not be useful as a tumour marker for Nigerians with hepatocellular carcinoma.

## Background

In most parts of the world, hepatic metastases are more common than primary malignant tumours of the liver although the reverse is true in sub-Sahara Africa and parts of the far East where hepatocellular carcinoma (HCC) is the most common malignant tumour of the liver [1]. Hepatocellular carcinoma is the sixth most common cancer in men and women combined with respect to the incidence rate [2]. Of the 12.7 million new cases of cancer diagnosed worldwide in 2008, hepatocellular carcinoma accounted for 5.9% (748,000) while of the 7.6 million cancer deaths worldwide in 2008, it accounted for 9.7% (1,234,000) [2].

Prevalence rate of HCC varies in different countries within Africa and it accounts for 12% of all malignancies in Nigeria [3]. The prognosis is poor due to late presentation with advanced disease. Early detection is still difficult due to lack of symptoms at the early stage and biomarkers that show high sensitivity and specificity.

Alphafetoprotein (AFP), is still the only serological marker presently available for routine screening in Nigeria and most parts of the world. Alphafetoprotein, discovered by Abelev in 1968 [4], is a foetal specific glycoprotein produced primarily by foetal liver, yolk sac and gut in the first trimester of pregnancy, but falls rapidly after birth in normal conditions.

Pathological elevation of AFP is seen in hepatocyte regeneration, hepatocarcinogenesis and embryonal carcinoma. Its limited utility in differentiating HCC from benign hepatic lesions and disorders such as acute exarcerbation of viral hepatitis has been a major drawback [5]. Novel tumour markers have been proposed for the screening of hepatocellular carcinoma. These include des-gamma carboxy prothrombin [5], Glypican 3, carbohydrate deficient transferin, Hepatitis B virus encoded x-antigen, α – L-fucosidase and recently cancer testis antigen [6]. These biomarkers except DCP, however lack adequate specificity to support a diagnostic value [5,7]. On the contrary, p53 antibody as a tumour marker is useful in predicting the grade and stage of HCC with respect to differentiation, proliferating activity and tumour progression [8].

Squamous cell carcinoma antigen (SCCA) is a new tumour marker recently discovered to be of diagnostic value in patients with hepatocellular carcinoma [9,10]. It is a serine protease inhibitor found physiologically in the spinous and granular layer of normal squamous epithelium of the skin. Increased levels have been found in epithelial cancers of the neck, cervix and lungs [9]. It is reported to be highly sensitive (84.2%) compared to 60% for AFP but has a low specificity (48.9%) for hepatocellular carcinoma [9].

However, Gianelli and coworkers at the University of Bari, Italy showed that a combination of AFP and SCCA yielded a correct diagnosis in 90.8% of patients with HCC [9]. In view of the high prevalence of HCC, the hopeless late presentation in our population, and the need to increase the diagnostic yield for HCC in Nigeria, a study assessing the importance of this serological marker in comparison to AFP for detection of HCC is desirable.

## Methods

Ethical approval for this cross sectional case- control study was obtained from the joint University College Hospital/University of Ibadan Ethical Review Committee.

Patients with clinical, sonographical and or histopathological confirmation of hepatocellular cancer admitted to gastroenterology and liver units of the University College Hospital, Ibadan were recruited for the study between October 2009 and October 2010 after informed consents were obtained. Patients with cancers other than primary liver cell cancer were excluded. Controls were sex matched apparently healthy relatives of patients with HCC. The ultrasound features were consistent with multiple intrahepatic tumours greater than 5 cm in 16 cases with no evidence of regional lymph node metastasis (T3a,N0,M0). Twenty- four cases were noted to involve multiple tumours with infiltration of the branches of the portal vein and regional lymph node metastasis (T3b,N1,M0) while intrahepatic masses with evidence of regional lymph nodes and distant metastasis were noted in 20 cases (T4,NI,M1) according to the AJCC TNM classification.

A total of 60 patients with hepatocellular carcinoma and 30 apparently healthy controls were recruited. Five milliliters of serum sample was taken from each patient and controls using aseptic technique and observing universal precautions. Blood samples were assayed for serum AFP, SCCA, HBsAg and Anti-HCV antibodies. Cut- off absorbance values were calculated according to manufacturer’s instructions and all the cases and controls with values greater than the cut- off were regarded as positive while those equal to or less than the cut- off were regarded as negative. Results were analyzed using the appropriate test for continuous variables and chi square test for analysis of quantitative variables.

Frequency tables, means, standard deviations, graphs and diagrams were used. Significant p value was taken to be less than 0.05. Sensitivity, specificity, positive and negative predictive values for each individual test were calculated while an ROC curve was used to compare the diagnostic accuracy of one tumour marker in relation to the other. AFP levels >200 mg/dl and SCCA levels >0.368 ng/ml were considered positive for HCC respectively in this study [9].

### Enzyme immunoassay for the quantitative determination of Afp and scca in human serum using scca and Afp Elisa Kit (diagnostic automation Inc, Canada)

The AFP Quantitative test kit is based on a solid phase ELISA. It utilizes one anti-AFP antibody for solid phase (microtitre wells) immobilization and another mouse monoclonal anti-AFP antibody in the antibody –enzyme (horseradish peroxidase) conjugate solution. Serum was added to the AFP antibody coated microtitre wells and incubated with the Zero Buffer. Human AFP if present in the serum combines with antibody on the well. The well was then washed to remove any residual test specimen. AFP antibody labeled with horseradish peroxidase (Conjugate) was added.

The conjugate bound immunologically to the AFP on the well resulting in the AFP molecules being sandwiched between the solid phase and enzyme linked antibodies. After incubation at room temperature, the wells were washed with water to remove unbound labeled antibodies. A solution of TMB was added and incubated for 20 minutes, resulting in the development of a blue colour.

The colour development was stopped with the addition of 2 N HCL and the colour changed to yellow. This was measured spectrophotometrically at 450 nm. The concentration of AFP is directly proportional to the colour intensity of the test sample.

For SCCA, the required number of micro plate strips was transferred to a strip frame and washed with the Wash solution. 25 μl of SCC Calibrators and serum sample was put into the strip well and 100 μl of antibody solution was added. After incubation at room temperature, each strip was washed six times before addition of TMB HRP – substrate to each well. This was incubated again for 30 minutes. The absorbance was measured spectrophotometrically at 620 nm.

## Results

There was male predominance among the patients, 40 being males (66.7%) compared to 20 (33.3%) females with a male to female ratio of 2:1. Similar values were obtained among controls. Ten (16.7%) of the cases were below the age of 30. Sixteen (26.7%) were between the ages of 30 and 39 years. Nine (5%) were within the 40–49 years age range. Thirteen (21.7%) were between 50–59 year age range while twelve (20%) were >60 yrs of age. The modal age range was between 30–39 years. There was no significant difference in the sex ratio of cases and control (p = 1.00) (Table 1).

**Table 1:** Age and sex distribution

**Characteristics**	**Cases (n = 60)**	**Control (n = 30)**	**Total (n = 90**	**X2 – value**	**P- value**
**1. Age group (years)**					
<30	10 (16.7)	14 (46.7)	24 (26.7)		
30-39	16 (26.7)	5 (16.7)	21 (23.3)		
40-49	9 (15.0)	2 (6.7)	11 (12.2)	10.275	0.036
50-59	13 (21.7)	3 (10.0)	16 (17.8)		
60+	12 (20.0)	6 (20.0)	18 (20.0)		
**2. Sex**					
Male	40 (66.7)	20 (66.7)	60 (66.7)	0.000	1.000
Female	20 (33.3)	10 (33.3)	30 (33.3)		

Of the 60 cases of HCC, 31 (51.7%) were positive for HBsAg as compared to six (20%) of the control group (p 0.004). Similarly anti HCV were present in 2 cases (3.3%) compared to 1 patient (3.3%) in the control group (Table 2).

**Table 2:** Prevalence of HBsAg & HCV among patients with HCC and control

**Characteristics**	**Cases (n = 60)**	**Control (n = 30)**	**Total (n = 90)**	**X**^2^**-value**	**P-value**
**HBsAg**					
**Yes**	31 (51.7)	6 (20.0)	37 (41.1)	8.284	0.004
**No**	29 (48.3)	24 (80.0)	53 (58.9)		
**HCV**					
**Yes**	2 (3.3)	1 (3.3)	3 (3.3)	0.000	0.743
**No**	58 (96.7)	29 (96.7)	87 (97.7)		

The mean AFP value for cases with HCC was 393.21 ± 386.97. This was considerably higher than the mean AFP level for the control group which was 5.60 ± 13.03 ng/ml (p value = 0.001). The range values for AFP in cases with HCC were between 1.68 ng/ml to >812.45 ng/ml which was the upper limit for concentration measurable by the kit used while the AFP range for control was between 0.43 – 72.95 ng/ml (Table 3).

**Table 3:** Comparison of AFP & SCCA cases and control

**AFP**	**Cases**	**Control**	**T-Value**	**P-Value**
Mean ± SD	393.21 ± 386.97	5.60 ± 13.03	5.455	0.001
Range	1.68-812.45	0.43-72.95		
**SCCA**				
Mean ± SD	0.64 ± 0.56	0.71 ± 0.65	−0.482	0.631
Range	0.15 – 3.56	0.02 – 2.65		

Thirty one of the cases (51.7%) had AFP values above 200 ng/ml. None of the controls had significantly elevated AFP levels. The sensitivity of the test was noted to be 51.7% while the specificity was 100%. The positive predictive value was equal to 100% and the negative predictive value was 49.2% (Table 4).

**Table 4:** Serum AFP in patients with HCC and controls (Levels > 200 ng/ml is considered positive)

	**Serum AFP Levels > 200 ng/ml**		**Serum SCCA levels > 0.368 ng/ml**		**Combination of serum AFP > 200 ng/ml and SCCA >0.368 ng/ml**	
	**Cases**	**Control**	**Cases**	**Control**	**Cases**	**Control**
Positive	31	0	45	22	26	0
Negative	29	30	15	8	34	30
Sensitivity	51.7%		75%		43.3%	
Specificity	100%		26.7%		100%	
Positive predictive value	100%		67.2%		100%	
Negative predictive value	49.2%		65.2%		53.1%	
Accuracy	67.7%		58.8%		62.2%	

SCCA sensitivity was noted to be 75% while the specificity was 26.7%. The positive predictive value was 67.2% and the negative predictive value was 65.2% (Table 4).

Only 26 patients with hepatocellular carcinoma had both positive AFP > 200 ng/ml and SCCA > 0.368 ng/ml. None of the controls had a combined AFP and SCCA value greater 200 ng/ml and 0.368 ng/ml respectively. A combination of positive AFP (>200 ng/ml) and positive SCCA (>0.368 ng/ml) increased the specificity to 100% (Table 4).

The AUC quantifies the overall ability of the test to discriminate between those individuals with the disease and those without. A perfect test with zero false positives and zero false negatives has an area of 1.00. The AFP screening test in this study has an AUC of 0.916 compared to SCCA with an area of 0.525 (Table 5).

**Table 5:** Area under the curve for quantifying discriminatory power of a test for those with and without HCC

**Test variables**	**Area**	**Std. error**	**P-value**	**95% confidence interval**
Alpha-fetoprotein	0.916	0.029	0.001	0.860-0.973
Squamous cell carcinoma antigen	0.525	0.069	0.700	0.389 – 0.661

The receiver operating characteristic curve (ROC) for alphafetoprotein is closer to the left hand border and the top upper left corner. This reflects its accuracy as a good screening test for hepatocellular carcinoma compared to squamous cell carcinoma antigen which is closer to the diagonal.

## Discussion

Alphafetoprotein values were frequently elevated in patients with HCC. AFP values >200 ng/ml was seen in 51.7% (31 cases) compared to controls. This is significantly lower than the 75% and 85% obtained by Alpert [11] and Fakunle [12]. It validates previous studies that have found that not all patients with hepatocellular carcinoma elaborate this tumour marker [5,7]. Values for SCCA were similar in both cases and control with no significant statistical difference (p = 0.631).

In this study AFP had an AUC of 0.916 which makes it a good screening test for the diagnosis of hepatocellular carcinoma. This was quite higher than the 0.717 found by Gianelli [9] in his own study which looked at the immunohistochemistry of liver tissues with hepatocellular carcinoma.

AUC for SCCA was noted to be 0.525 with a 95% confidence interval of 0.389-0.661. This study did not find a significant level of discriminatory power of SCCA between HCC patients and healthy control groups unlike previous studies [9,10]. This may be explained by the fact that previous studies involved tissue specimens of both primary nodules of HCC and peritumoural areas. They performed Immunohistochemistry quantification on tissue specimens. SCCA antigen appeared strongly stained in tumoral tissues [9,10]. However, no correlation was observed between serum and tissue levels of SCCA antigen (r = −0.094, p > 0.1) [9,10]. SCCA is mainly expressed in cytosol and is not associated with membrane bound organelles, therefore circulating serum levels are likely due to cell lysis rather than a secretory process. This may account for why serum SCCA may not be ideal discriminatory marker for HCC diagnosis.

SCCA sensitivity was 75%, compared to AFP sensitivity of 51.7%. It compares favorably with work done by Gianlugi Giannelli *et al*[9] where SCCA sensitivity was 84.2%. However the specificity of SCCA in our study was poor (26.7%), compared to AFP specificity of 100%. The specificity was also poor in the study by Gianluigi Gianneli (48.9%). This antigen is noted to be common in the skin, saliva and sweat of apparently normal people and this may account for the values that were found in the controls.

There are therapeutic techniques for the treatment of hepatocellular carcinoma in the early stage; hence, emphasis is now being placed on early detection in patients at risk who are presently asymptomatic. Detection of HCC at an early stage may reduce mortality significantly. Mass screening may be justified as the population at-risk can be identified easily and tumour ablation or resection at an early stage can increase survival. However, massive screening should be justified only when sensitive and specific diagnostic procedures are available.

Des- gamma- carboxy- prothrombin (DCP) is another promising tool with limited expense and wide accessibility. A bivariate meta- analysis of the diagnostic performance of DCP by Gao et al [5] found a sensitivity of 67%, specificity of 92%, positive likelihood ratio of 92% and an AUC of 0.89. They however noted significant heterogeneity and recommended more prospective studies. DCP assay was not one of the objectives in this present study, however, it is recommended for future considerations.

Presently, SCCA is still a research tool and has not been approved for widespread clinical use in the screening of patients with hepatocellular carcinoma. It is also very expensive. Unfortunately as shown in this study, it also lacks good discriminatory power between with HCC and apparently healthy controls and may not be useful in this subset of patients. We noted that all the cases recruited into this study presented with advanced hepatocellular carcinoma and died within 3 months of presentation, reflecting the poor prognosis at this stage. Present recommendation focuses on 6 monthly ultrasound surveillance and measurement of serum Alphafetoprotein in patients with chronic hepatitis and or cirrhosis.

Thirty one cases (51.7%) were found to be HBsAg positive in this study. This represents a significant association between HCC and HBV as an aetiological agent in this study.

Previous study by Ndububa [13] noted a prevalence of 61% of HBsAg in HCC. Our study did not find a significant difference between HCV infection in cases and control. Only 2 cases and 1 apparently healthy control were positive for HCV. While the risk was not significant in this study, it has however been well proven that HCV is an important aetiological risk factor for the development of hepatocellular carcinoma. This is especially true in Western Europe and Asia [14-16].

Van Roey [16] found that 55% of HBsAg negative cirrhotic patients with HCC were anti-HCV positive. The reduced prevalence found in our study suggests that HCV infection may not be a strong risk factor compared to hepatitis B in our patients.

More work need to be done in search of appropriate tumour markers for early detection of hepatocellular carcinoma among Nigerians.

The scope of this study was limited majorly by funds. However, future studies will consider inclusion of controls with cirrhosis and metastatic liver disease. Another major limitation was the inability to study the efficacy of SCCA in patients with early HCC due to late presentation in our environment. This is reflected in our study where all the patients presented with advanced HCC.

## Conclusions

We conclude that AFP remains a relatively good screening marker for HCC in Nigerians and SCCA lacks accuracy as a screening test for its diagnosis in Nigerians. Hepatitis B remains a strong aetiological agent of liver cancer in Nigeria and efforts at childhood immunization will help in reducing the disease burden.

## Competing interests

The authors of this article hereby declare that they have no competing interests.

## Authors’ contributions

Dr Soyemi Olufemi: Conceptualization and design of study, Drafting of Article. Dr Otegbayo Jesse Abiodun: Critical Review of Article for intellectual content. Dr Ola Samuel Olawale: Manuscript Review. Dr Akere Adegboyega: Literature Search. Dr Soyemi Temitope: Manuscript preparation, acquisition of Data. All authors read and approved the final manuscript.
